# Patent Instruments

**Published:** 1860-12

**Authors:** J. A. Murphy


### PATENT INSTRUMENTS.

BY J. A. MURPHY, M. D.

The propriety of obtaining a patent for a surgical instrument, or appliance, is no longer a matter of discussion among educated gentlemen of the regular medical profession. There is a small class, however, who by no means are liberally educated, or appreciate their profession as a liberal one, who occasionally procure a patent for some imaginary new instrument or appliance. These patented instruments meeting with no favor in the regular profession, are soon forgotten, or are withdrawn from public notice. In most cases in our knowledge, the physician who obtains a patent for a supposed new, useful and wonderful instrument is ruined in practice, and professional reputation. He becomes so absorbed in his pet, that he neglects his business, and travels about from place to place, advertising his instrument in violation of the Code of Ethics of the American Medical Association. What says the Code? In art. 1, sec. 4, we find the following language: "*Equally derogatory to professional character is it, for a physician to hold a patent for any surgical instrument or medicine; or to dispense a secret nostrum, whether it be the composition or exclusive property of himself or others. For, if such nostrums be of real efficacy, any concealment regarding it is inconsistent with beneficence and professional liberality; and, if mystery alone gives it value and importance, such craft implies either disgraceful ignorance or fraudulent avarice. It is also*

*reprehensible for physicians to give certificates attesting the efficacy of patent or secret medicines, or in any way to promote the use of them.*" This is not alone the doctrine of the profession of our own country, but it is also of the British, German and French profession. The medical profession is a liberal beneficent one. Its great first cardinal motive is to do good to humanity. In accomplishing this great and holy end, we are a band of brethren claiming the assistance of each other. The knowledge of the profession is common property. Whatever any man may discover that in any way tends to prevent, palliate or cure disease, he is bound to lay on the common altar, if he claims to be a member of a profession, the pages of whose history are filled with the deeds of men whose whole lives have been devoted to the physical relief of their race. At the present time, and, indeed, in all ages, the good and the true physician, the liberally educated gentleman, is publishing his observations of disease—his experience with new remedies—his opinions as to the causes and prevention of disease, so that all may have the benefit. What leads him to this course? Nothing else than that his brethren all round the wide world may use them in the treatment of their fellow-men. His reward is in doing good. The difference between laying down a great law for the treatment of a disease, and the invention of a new instrument, is difficult to be made out. As well might he who experiments with a new remedy claim a patent as he who invents a new instrument. We have given one of the great objections to patent instruments. The other, and in our country the greatest, is that it implies " disgraceful ignorance or fraudulent avarice," and is the habit of quacks of all kinds.

It is the unerring sign of a quack, ignorant of the history of his profession, wanting in the tone, spirit and manners of a gentlemen, to obtain patents for secret remedies and surgical instruments, and boast that he has certain knowledge or a particular instrument which no one else in the profession possesses. It is rare to find a regularly educated physician guilty of such conduct. There is still another reason, and a strong one, which holds against patented instruments. It is that the profession which requires such a high order of preliminary education, and such long and incessant study for its mastery, is brought down on a level with the every day trades and callings of life. The professional man who patents his instruments is leveled with the man without education, without study, who invents a lock, a churn or cider-press. His great idea in seeking to patent his churn or cider-press, is not to benefit his race, but to put money in his pockets. His desire is to put money in his pockets,—benefit to humanity is another consideration. His cardinal principle is to succeed—to make gain, without any respect to his race, or those who may be pursuing the same trade.

Has this been the course of the distinguished and good physician in any age? The great Webster once said, every man is a debtor to his profession. Every true man willingly acknowledges this debt, and strives to repay it by giving over the best results of his labors.

Let every man, then, whose genius may originate a new remedy, or whose generalization of a large number of well-observed facts may enable him to enunciate a new law, or who may contrive a new instrument, freely and generously give them to his professional brethren without money and price, and without the restrictions of a patent. Not only will his profession honor him, but humanity will call him blessed. In this course is the profession to be kept above quacks, and all those who regard it as a trade. It should never be forgotten that the profession, and the world at large, gives the full meed of praise to all who are true, generous benefactors of their race. It is a fact, we believe, that many of those instruments which now bear the inventors' names, were never patented, and it is no less true, that of the various surgical instruments and appliances which have been patented in our country in the last twenty years, the fewest number are even now heard of, much less used. There is no practical difference or distinction between those who patent a pill, a balsam or a plaster, and those who patent an instrument. It is disreputable, unprofessional, and opposed to the acquirements of all who attain to the position of gentlemen, good physicians, and scientific men. Who are the men whom every now and then we meet with or read of, who have patented some miserable fracture-bed, or an instrument by which every man may extract his own teeth, or a painless and harmless pain-killer, or a cough syrup which will cure every cough! Need we say they are sheer adventurers, traveling mountebanks, and persons of the most shallow pretensions. It is well, then, for every man to know, that he can have no position in the regular profession, or be acknowledged in medical society as a gentleman, if he procures a patent for an instrument. He is a quack, and a quack he must remain. He is ranked with the class of so-called Eclectics, Homeopaths, Spiritual and Water Doctors, and with that class of advertising scoundrels, who fill the newspapers with lying advertisements; with that class who advertise to a credulous and ignorant public, that after years of study (!) they can cure certain diseases, without the knife, or any medicine; or with that class of swindlers who practice all systems, according as their patients may desire. There is but one standard of respectability in every profession. For our profession we have the Code of Ethics, which forbids the procuring, holding, using or attesting to the benefits of patent instruments, and all those who are gentlemen, and friends of a dignified, learned, and time-honored profession, will give their cordial support to the Code in letter or spirit; quacks have no code, and especially despise it, for the reason that it draws a broad line between them and the members of the legitimate profession.

Remarks.—The principles alluded to in the above article are equally as applicable to each department of medical and surgical practice, as to the whole, and the same arguments are operative in their application to the practitioner of a specialty, as to the general practitioner. The correctness of this is apparent to all. True medical men maintain everywhere that this is a correct principle.

The practitioner of specialties have, as a general thing, been in bad repute, with the regular medical profession. It is true there are, and always have been, some noble exceptions. This want of affiliation of the general for the special practitioners has originated chiefly from two causes. The first is a want of genuine, scientific and professional knowledge. The great body of specialists have been little else than ignorant pretenders. The second is, that the specialists have usually indulged in practices which have been accounted unprofessional and illegitimate. No one should expect to be received in full fellowship, or even recognized, while pursuing such a course.

That specialists have, as a general thing, been ignorant of the fundamental principles of medical science, without which they are totally unfit to practice in any department, their history abundantly proves ; and that they have ever been doing that which is accounted unprofessional and illegitimate is as clearly proven by their history. I propose a few remarks on only one particular of this subject, viz : that which is referred to in Dr. Murphy's paper—" the procuring patents on anything pertaining to the profession."

The dentist, who advocates patents for anything pertaining to his profession, arrays himself against the opinions and judgment of the best and most intelligent medical practitioners of this and other countries, and at once forfeits their regard for and appreciation of him as a professional man; which would be gratifying, at least, to every true man to possess. This loss always far more than counterbalances all that is gained by the procurement or advocacy of patents. The principle upon which the medical profession repudiates patents, we doubt not, is pretty fully understood by all, at least, by all intelligent dentists. The extra tax or cost of that which is patented, where it is intimately connected with the relief of human suffering, constitutes one strong objection toprofessional patents.

Nothing should be done, to place that which is designed for the relief of disease, out of the reach of any. What would have been thought of Jenner if he had hedged about the discovery of vaccination with a patent, so that it could not be used without paying him a bonus? He would have received, and merited too, the burning indignation of a civilized world. He might have made all the pleas now made in justification of a patent, he might have argued that it would be a great benefit to mankind, that they would receive far more than it would cost them, even though they did pay a patent fee; that he was poor and needed the money, and a great many people would use it who were abundantly able to pay; that he had expended time, money and labor in studying those things, which brought about this result. Would the world, to say nothing of the medical profession, receive as valid, such excuses for patenting the process of vaccination.

Every argument that can be adduced in favor of dental patents, will sustain with equal force, in any and every particular, patents in the medical profession. If one medical man may obtain a patent and deal in them, another may, so then all, both the high and the low, the humble and the mean may become hucksters in patents. One great and almost universal argument, brought forward in support of dental patents, is that every one should be rewarded for expenditure of time, money and energies; if a person spends his time, money and energies, aye, even racks his brain, and produces nothing,how shall he be paid and who will do it: he has done the same as another, and with just as good a motive too, but the result has not been the same, nothing has been produced: now, does any body propose to pay the man who has expended much time, large capital and great energy, simply because he has done so: the man is yet to be found who has acted upon that principle. Again, as an evidence that there is nothing in that kind of an argument, those who are disposed to buy patent articles, do so because of some supposed value in the thing itself, independent of the investment of time, money and energy, which the inventor may have made. Men often pay as much, or more, for a patent, the whole plan of which flashed into the mind in ten seconds of time, as for those that cost years of toil and study. The thing accomplished is that which is taken into consideration, and not the manner of its accomplishment.

Again, it is maintained that it is the best manner of disseminating a good invention. This is fallacious. Anything that has real merit, will be disseminated, whether patented or not. Would vaccination have been more generally employed if it had been patented; and so with any other really valuable process, instrument or appliance. The tendency of a patent is to restrict the use of that to which it pertains, and for two reasons; the feeling of opposition that exists with so many physicians and dentists against a patented article or process, prevents them from obtaining or using it; and again, the additional tax imposed by a patent prevents many from employing the instrument or appliance to which it pertains.

The history of dental patents shows a fearful record of disappointed and broken down inventors, one that should, we think, be quite sufficient to discourage any one who contemplated procuring or dealing in dental patents. Three-fourths of the instruments or processes, for which patents have been obtained, are without merit, and consequently require a great pressing, to make them amount to anything. Inventors usually see more in their inventions than any one else, and generally anticipate great gain from them, and so they abandon the practice of the profession to engage in pressing the claims of their invention, and in vending it. This, generally, results in great loss, and not unfrequently in complete ruin, pecuniarily. Many examples of this kind in the dental profession will occur to the mind of every one.

Not one in twenty who obtains dental patents ever makes money by them, and every one who does so loses cast with his profession, and that, just in proportion to the elevation of the position which he occupies.

Now, if the dental is a branch of the medical profession, and seeks to be recognized as such, and if obtaining or dealing in patents is in opposition to the feelings, the judgment and the code of ethics of the medical profession, and since such a course is always derogatory to professional standing, and in almost all cases attended with pecuniary loss, is it not better to ignore the whole matter of dental patents, cast them away, and have nothing to do with them. It is said that men should be rewarded for their valuable inventions. To this we have no objections, but the record shows that patenting an invention is not a money-making operation to the inventor at least.

If any one, in his great liberality, desires to reward an inventor, he can do it without buying a patent. Those in the medical profession, who have made valuable discoveries and given them freely, have received much more in the way of honor, and pecuniary reward too, than those who have pursued a hedging, exclusive course.

There seems to be a disposition upon the part of some to make a distinction between patenting an instrument or appliance and a process. Practically and in effect we can see no difference. It is quite as proper to patent the process of extracting a tooth (if it were possible) as to patent the instrument with which that operation is performed; it is just as legitimate to patent a particular method of filling a tooth, as to patent the instrument with which that operation is performed. The chief moving inducement in procuring and dealing in patents is gain, it is for self, and not for the good of mankind, and in this respect it is greatly at variance with the profession of medicine, in all its branches, the glory of which is that it has for its chief and great object good to humanity, relief from suffering, deliverance from disease, that fell destroyer of the human kind. T.

